# New ways of working and intrapreneurial behaviour: the mediating role of transformational leadership and social interaction

**DOI:** 10.1007/s11846-020-00412-1

**Published:** 2020-09-22

**Authors:** Ruud Gerards, Sanne van Wetten, Cecile van Sambeek

**Affiliations:** 1 Research Centre for Education and the Labour Market (ROA), School of Business and Economics, Maastricht University, Tongersestraat 49, 6211 LM Maastricht, The Netherlands; 2 School of Business and Economics, Maastricht University, Tongersestraat 49, 6211 LM Maastricht, The Netherlands

**Keywords:** Intrapreneurial behaviour, Employee entrepreneurship, New ways of working, Transformational leadership, Social interaction, 90B50, 91B39

## Abstract

To promote innovativeness and efficiency, an increasing number of firms have adopted New ways of working (NWW). However, it is not clear what effect NWW has on innovation-related outcomes, such as intrapreneurial behaviour. Therefore, we provide a first investigation on the relation between the facets of NWW and intrapreneurial behaviour, while testing transformational leadership and co-worker social interaction as mediators. We use a sample of 254 employees of the Dutch working population and apply the Preacher and Hayes’ (Behav Res Methods 40(3):879–891, 2008) bootstrap method for multiple mediation to test our hypotheses. We find that NWW facets time- and location-independent work and management on output are positively related to intrapreneurial behaviour. In addition, we find that the relation between a freely accessible open workplace and intrapreneurial behaviour is mediated by transformational leadership. However, we find no relationship between co-worker social interaction and intrapreneurial behaviour and thus no mediating role for social interaction. Our research adds to the budding literature on the effects of NWW and to the literature on the determinants of intrapreneurial behaviour. We conclude that implementation of several NWW facets and a transformational leadership style could help foster intrapreneurial behaviour among employees, and that future research that further enhances the measurement of the NWW facets and investigates its configurational effects on intrapreneurial behaviour is welcome.

## Introduction

Innovation and entrepreneurship are crucial aspects of business success and societal development (Cefis and Marsili 2006; Kraus et al. 2012; Mumford 2000). An important source of innovation stems from bottom-up entrepreneurial activities by employees (Rigtering and Weitzel 2013; Rigtering et al. 2019; Sharma and Chrisman 1999). These entrepreneurial employees are known as intrapreneurs (Blanka 2018; Pinchot 1985). Intrapreneurs instigate new internal ventures and strategic renewal and enter new markets on behalf of their employers (de Jong et al. 2015; Gawke et al. 2017, 2019). Organisations that wish to stimulate their employees to engage in intrapreneurial behaviour should be organised in a way that promotes openness to innovation and entrepreneurship (Kuratko et al. 1990, 2014; Mustafa et al. 2018). To promote innovation and efficiency, an increasing number of organisations have implemented New ways of working (NWW) (Bijl 2009; Brunia et al. 2016; Kingma 2018; Nijp et al. 2016). Studying a sample of 254 employees across various economic sectors and occupations in the Netherlands, this empirical study provides a first investigation as well as a path forward into the relationship between NWW and intrapreneurial behaviour.

NWW are a bundle of human resource management practices that affect employees’ physical workspace, technology, organisation and management, and the work culture (Blok et al. 2012; Gerards et al. 2018). Gerards et al. (2018, forthcoming) distinguish five NWW facets. First, *time*- *and location*-*independent work*, that allows employees to work anytime and anywhere they want, facilitated by information and communications technologies (ICT). Second, *management of output*, which implies that instead of the monitoring of work processes, employees are managed according to their performance and output. Third, *access to organisational knowledge*, enabled by ICT that offers unlimited accessibility and connectivity to colleagues and organisational networks. Fourth, *flexibility in working relations*, entailing the freedom for employees to prioritise and organise their employment relation in such a way that suits their personal situation, ambition and lifestyle (Gerards et al. 2018). Fifth, a *freely accessible open workplace*, such as an open-plan office, designed to foster collegial encounters. In sum, NWW aim to grant employees more flexibility, autonomy and freedom with the support of electronic communication (Peters et al. 2014). We focus on the effects of the individual NWW facets, since they can be implemented independently from one another.

Given the increasing number of organisations that adopt one or more facets of NWW, it is remarkable that there is only limited understanding of its effect on employee outcomes (Blok et al. 2012; Nijp et al. 2016; Gerards et al. 2018). Recent studies show, however, that NWW affect employee outcomes, such as informal learning efforts (Gerards et al. forthcoming) and work engagement (Gerards et al. 2018). The recent COVID-19 induced increase in working from home (e.g. Brynjolfsson et al. 2020)—which utilizes the first three NWW facets—underscores the relevance to learn more about the employee outcomes of NWW.

We build on social exchange theory (Blau 1964; Emerson 1976; Homans 1958) and theorise that NWW affect intrapreneurial behaviour by altering the social exchanges between the organisation and its employees, between individual employees, and between employees and their managers. Using social exchange theory helps us form expectations about the relations between NWW and employee intrapreneurial behaviour, because these relations do not follow unambiguously from prior literature. For instance, recommendations from the literature on facilitating intrapreneurship often focus on increasing the NWW related job resources flexibility, autonomy and freedom (e.g. de Jong et al. 2015; Kuratko et al. 1990, 2014; Martins and Terblanche 2003). However, the positive relation between for example autonomy and intrapreneurial behaviour is not unambiguous. On the one hand, autonomy (as part of NWW) entails the absence of direct supervision and a focus on outputs that demands and stimulates a transformational leadership style (Gerards et al. 2018), which in turn is known to facilitate intrapreneurial behaviour (Moriano et al. 2014). On the other hand, in fear of negative reciprocal consequences, employees will be reluctant to show initiative when organisations and leaders emphasise efficiency and flawlessness, even when the employees are given autonomy (Jung et al. 2003; Yukl 2001).

This example points to the important role managers and their leadership behaviours play in facilitating innovation and entrepreneurship in their organisations (Mueller et al. 2018; Sperber and Linder 2018). Previous research shows that managers’ leadership styles influence employees’ intrapreneurial behaviour (e.g. Edu Valsania et al. 2016; Moriano et al. 2014). For instance, Edu Valsania et al. (2016) show that authentic leadership is positively related to intrapreneurial behaviour. In addition, Moriano et al. (2014) show that transformational leadership positively relates to intrapreneurial behaviour, whereas transactional leadership does not.^1^ Interestingly, Gerards et al. (2018) elaborate how NWW may actually help push a managerial change towards transformational leadership. These findings suggest that NWW may relate to intrapreneurial behaviour via an increase in transformational leadership. However, it has not yet been studied empirically how managers’ leadership styles and human resource practices such as NWW—together—relate to employees’ intrapreneurial behaviour. Therefore, we include transformational leadership as potential mediator in our analysis of the relation between the NWW facets and intrapreneurial behaviour.

Organisational networks are another known facilitator of intrapreneurial behaviour (e.g. Heinze and Weber 2016; Rigtering and Weitzel 2013), because the social exchanges that occur in these networks facilitate the development of novel ideas (Bouncken et al. 2018; Fried et al. 2007; Russell 1999) and concomitantly employee intrapreneurship. However, employees’ flexibility in work location and time of day, in number of working hours, and the increased reliance on ICT entailed in NWW, can simultaneously make for obstruction and facilitation of social exchanges (Gerards et al. 2018). High quality social exchanges between co-workers are characterised by mutual support and high levels of interactions (Fried et al. 2007). Therefore, we use the concept of ‘social interaction’ to capture these relations between co-workers (Gerards et al. 2018), and we employ it as another potential mediator in our analyses.

Our first contribution is to the literature on the effects of NWW, by providing a first investigation of the relation between the NWW facets and employees’ intrapreneurial behaviour. In addition, we refine the measurement of the NWW facets, which can be used to further develop the NWW measurement scale. Our second contribution is to the literature on the determinants of employees’ intrapreneurial behaviour. Previous studies show for instance that organisational structure (Kuratko et al. 2014) and job design (e.g. de Jong et al. 2015; Rigtering and Weitzel 2013) affect employees’ intrapreneurial behaviour. We add to this body of research by investigating the contribution of NWW facets to employees’ intrapreneurial behaviour, while controlling for individual-level determinants of intrapreneurial behaviour. We find that particularly the NWW facets that entail granting employees autonomy in choosing when, where and how to work, relate positively to intrapreneurial behaviour, and that the relation with open workplaces is positively mediated by transformational leadership.

## Literature review and hypotheses

### Intrapreneurship and intrapreneurial behaviour

Intrapreneurship is generally described as entrepreneurship within an existing organisation (Blanka 2018; Menzel et al. 2007) and intrapreneurs are often described as entrepreneurial employees (Antoncic and Hisrich 2001; Blanka 2018; Pinchot 1985). Although intrapreneurship refers to entrepreneurial behaviour by individual employees,^2^ research on entrepreneurship within organisations has mainly focused on entrepreneurship at the organisational level (Blanka 2018), which is known as corporate entrepreneurship (Antoncic and Hisrich 2003; Covin and Slevin 1991; Guth and Ginsberg 1990; Lumpkin and Dess 1996; Zahra 1991). The term corporate entrepreneurship is used to describe a process that leads to renewal, innovation or the creation of new organisations in an existing organisation (Blanka 2018; Sharma and Chrisman 1999). Intrapreneurship and corporate entrepreneurship are distinct but related phenomena. Corporate entrepreneurship can be seen as a top-down initiated process within organisations, whereas intrapreneurship is seen as a bottom-up process initiated by employees (Blanka et al. 2018; Rigtering and Weitzel 2013). However, these processes are symbiotic, as both self-initiated employee behaviour and organisational antecedents are needed to observe employees’ intrapreneurial behaviour (Blanka 2018; Åmo and Kolvereid 2005). As such, corporate entrepreneurship can influence the instigation of employees’ intrapreneurial behaviour and vice versa (Blanka 2018).

Intrapreneurial behaviour can be conceptualised in several ways. Intrapreneurial behaviour is usually conceptualised as employees’ innovative, proactive and risk taking behaviours (e.g. de Jong et al. 2015; Rigtering and Weitzel 2013; Stam et al. 2012). However, from this conceptualisation it remains unclear what is specifically intrapreneurial about these behaviours and how they lead to entrepreneurial outcomes. Therefore, Gawke et al. (2017, 2019) conceptualise that what sets intrapreneurs apart from other innovative and proactive employees, are behaviours that specifically revolve around new venture creation and strategic renewal. New venture creation involves the development of new businesses or new organisations (Gartner 1985). New venture behaviour consists of an employee’s agentic and anticipatory behaviours with the goal of creating new business or new organisations for an existing organisation (Gawke et al. 2017, 2019). Strategic renewal entails the process, content and outcome of attributes of an organisation being refreshed or replaced, with the potential of affecting long-term prospects (Agarwal and Helfat 2009). Strategic renewal behaviour includes behaviours that aim to increase an organisation’s ability to react to internal and external developments (Gawke et al. 2017, 2019).

A growing body of research investigates entrepreneurship at the employee level and attempts to uncover the organisational and job characteristics that drive intrapreneurial behaviour (e.g. de Jong et al. 2015; Rigtering et al. 2019). The organisational context has been found to play an important role in facilitating or obstructing intrapreneurial behaviour (Blanka 2018; Mustafa et al. 2018; Neessen et al. 2018). The organisational context of intrapreneurs allows them to make use of organisational resources and networks, and enjoy support and flexibility in case of failure (Blanka 2018; Zenovia 2011). However, the organisational context can also entail restrictions with regard to autonomy and flexibility as they are subject to existing policies, regulations and bureaucracy (Zenovia 2011). Moreover, in contrast to entrepreneurs, employees’ contracts usually translate into limited potential rewards for intrapreneurial behaviour. However, as the ownership of the idea and the inherent financial risk of pursuing an entrepreneurial opportunity remain with the organisation, employees (unlike entrepreneurs) are shielded from these and instead carry career related risk in case of project failure (Kacperczyk 2012).

### New ways of working

New ways of working are a recent phenomenon that arguably started with a speech and white paper by Bill Gates in 2005 titled ‘The New World of Work’^3^ (Bijl 2009). NWW have been enabled by rapid advances in ICT—notably the internet (Bijl 2009)—and aims to simultaneously recruit and retain a diverse workforce that increasingly varies in preferences, while boosting innovation and productivity, and cutting costs (Nijp et al. 2016). It consists of a bundle of HRM practices enabling employees to work independent of time and place, supported by a flexible work environment that is facilitated by ICT (de Leede 2016). NWW grant employees more freedom and autonomy (Peters et al. 2014) through five facets distinguished by Gerards et al. (2018, forthcoming) based on their overview of the literature: (1) time- and location-independent work, (2) management of output, (3) access to organisational knowledge, (4) flexibility in working relations, and (5) a freely accessible open workplace. Note that each of the five NWW facets can be implemented independently in an organisation. We now provide a brief explanation of these five facets and refer to Gerards et al. (2018) for more details.

*Time*- *and location*-*independent work* constitutes working independently of time and location. Employees are not bound by a work schedule and as such have a higher level of autonomy in choosing when and where to work. *Management of output* relates to the management of employees and their work processes. As office presence is no longer required, management focusses on acquired results and output rather than how employees organise their work. The latter is left to the discretion of the employee. Modern ICT systems enable the facet *access to organisational knowledge*, which entails that employees have access to all knowledge in the organisation and can easily and quickly connect, communicate, and share their own knowledge with colleagues and supervisors. *Flexibility in working relations* relates to workers’ influence over their work-life balance, for which employees might have different preferences depending on for instance their career stage or household structure (Darcy et al. 2012; ten Brummelhuis and van der Lippe 2010). This facet allows employees to tailor their employment relation in a way that suits their private situation with regard to household and family activities, lifestyle or professional ambition. The *freely accessible open workplace* entails open-plan offices designed in such a way that they encourage communication and cooperation by fostering encounters between colleagues (Gerards et al. 2018).

### New ways of working and intrapreneurial behaviour

We argue that the facets of NWW affect intrapreneurial behaviour by altering the social exchanges between the organisation and its employees, between employees and their managers and amongst employees. Social exchange theory (SET) states that social exchanges are mutually reinforcing (Homans 1958). Because employee behaviour is motivated by the rewards they expect to gain from their behaviour (Blau 1964), positive social exchange relationships motivate employees to engage in further behaviours that reinforce those positive relationships (Cropanzano and Mitchell 2005; Hughes et al. 2018), as this results in socio-emotional and/or economic rewards.

Social exchanges are sustained by trust, which can facilitate actions that entail risk taking such as intrapreneurial behaviour (Hughes et al. 2018; Mayer et al. 1995; Rigtering and Weitzel 2013). As intrapreneurial behaviour entails career related risk for the employee (Kacperczyk 2012), employees are likely to avoid these risks (Colquitt et al. 2011) and forego intrapreneurial behaviour if they don’t feel trust in the organisation. In addition, the reciprocity principle (Blau 1964; Cropanzano and Mitchell 2005; Homans 1958; Meeker 1971) entails that positive social exchange relationships urge employees to reward these relationships by performing their tasks in new and better ways, triggering innovative behaviours (Bednall et al. 2018; Hughes et al. 2018; Scott and Bruce 1994). Therefore, we theorise that the implementation of NWW signals an organisation’s trust in their employees to execute job tasks effectively, even while working at a time and location of their own choosing and in the absence of direct supervision. In turn, employees may reciprocate and reward this positive social exchange by behaviours that are aimed at creating a positive impact for the organisation, such as intrapreneurial behaviour.

Next, we discuss the empirical evidence for the relation between NWW and intrapreneurial behaviour per facet. The first NWW facet, time- and location-independent work, grants employees autonomy in when and where to work. Autonomy is defined as “the degree to which the job provides substantial freedom, independence, and discretion to the individual in scheduling the work and in determining the procedures to be used in carrying it out” (Hackman and Oldham 1976, p. 258). Time- and location-independent work relates mainly to employees’ autonomy in scheduling and as such relates naturally to time availability, which is the degree to which jobs are structured and schedules are created in the workplace (Kuratko et al. 2014). Time availability is crucial for employees to explore and realise new ideas (Deprez et al. 2018; Kuratko et al. 2014; Puech and Durand 2017; Rigtering and Weitzel 2013). For instance, Puech and Durand (2017) find that intrapreneurial ideas and opportunities are mostly identified during daily tasks. However, when employees do not have time available for the exploration and development of new intrapreneurial projects, these projects are likely to fail before they even go beyond the idea phase. Therefore, Puech and Durand suggest that especially the exploration of the value of new ideas requires autonomy for employees to organise part of their work themselves. Accordingly, we argue that the autonomous and flexible scheduling that is associated with the NWW facet time- and location-independent work, may facilitate employees to allocate time to intrapreneurial activities and as such may facilitate intrapreneurial behaviour. However, as working outside the office has been shown in some studies to negatively affect social exchange relationships,^4^ time- and location-independent work could also hinder intrapreneurial behaviour. However, Gerards et al. (2018) in a comparable context to this study, do not find any significant relation between the NWW facet time- and location-independent work and social interaction. Therefore, we argue that the potential negative influence on intrapreneurial behaviour of time- and location-independent work via social exchange relationships—if present at all—is probably outweighed by the positive influence of time availability. We formulate the following hypothesis:

#### H1a

Time- and location-independent work is positively related to intrapreneurial behaviour.

The second NWW facet, management of output, is also likely to foster intrapreneurial behaviour. Employees’ intrapreneurial behaviour is facilitated when an organisation provides employees with work discretion, i.e. provides employees with freedom in the way in which they do their work, refutes excessive supervision and allows for decision making freedom (de Jong et al. 2015; Kuratko et al. 2014). The facet management of output is therefore likely to foster intrapreneurial behaviour by allowing for procedural and decision-making autonomy. By managing output rather than input and focussing on results rather than processes, managers can grant employees the autonomy they need for intrapreneurial behaviour. Managers have to support intrapreneurs, not manage their processes, and provide them some leeway for changes (de Jong et al. 2015; Fry 1987). In addition, less management control helps the communication of intrapreneurial ideas (Deprez et al. 2018). We therefore expect that:

#### H1b

Management of output is positively related to intrapreneurial behaviour.

The third NWW facet, access to organisational knowledge, is likely to support the creative process of intrapreneurs. Freely accessible information is one of what Kanter (1985) describes as the ‘power tools’ in facilitating intrapreneurial behaviour. By providing employees with access to all organisational knowledge, the flexibility of organisational boundaries is increased, which enhances information flows between individuals, departments, the organisation and the external environment (Kuratko et al. 2014). In addition to providing access to knowledge, ICT applications often support the development of organisational knowledge networks by facilitating interactions between team members, managers and colleagues from other departments. These knowledge networks increase employees’ access to new perspectives and insights, helping them to generate new ideas by combining information and knowledge from various sources (Perry-Smith 2006; Perry-Smith and Shalley 2003)—an essential element of intrapreneurship (Hayton and Kelley 2006). Therefore, we formulate the following hypothesis:

#### H1c

Access to organisational knowledge is positively related to intrapreneurial behaviour.

The fourth NWW facet, flexibility in working relations, concerns the adaptability of working hours and as such adds another layer of flexibility of how much to work. Both the mere availability and the actual use of this flexibility have been shown to relate positively to work engagement (Bal and De Lange 2015), which in turn is positively related to intrapreneurial behaviour (Gawke et al. 2017). Moreover, if this flexibility is used to temporarily work more hours, it may help employees develop intrapreneurial projects (Puech and Durand 2017). However, the common use of this flexibility is to reduce one’s workload in response to personal or family circumstances (e.g. caring for a family member) or when personal preferences tend more towards leisure (Bal and De Lange 2015). As intrapreneurial behaviour is known to relate positively to the number of hours worked (Adachi and Hisada 2017), the reduced workload commonly associated with flexibility in working relations may decrease intrapreneurial initiatives. However, since we operationalize this facet in terms of its availability and not in terms of its actual use (see Sect. 3.2.2), we expect that flexibility in working relations in this context relates positively to intrapreneurial behaviour.

#### H1d

Flexibility in working relations is positively related to intrapreneurial behaviour.

We also expect that the fifth NWW facet, a freely accessible open workplace, is positively related to intrapreneurial behaviour. The design of the workspace impacts the behaviour of its users, and open-plan offices are specifically designed to foster communication and collaboration (Elsbach and Bechky 2007). Although open-plan offices can also be experienced to disturb the work process, there is a widespread belief that open-plan offices facilitate social exchanges (Bouncken et al. 2018; Kim and de Dear 2013). Moreover, the physical work environment is known to influence employees’ creativity (Dul et al. 2011). Open-plan offices in particular, foster employee creativity by increasing communication and interaction (Bouncken et al. 2018; Lewis and Moultrie 2005). The informal setting provided by freely accessible open workplaces also provides employees with opportunities to mention and discuss ideas casually, lowering the perceived risk of voicing a new idea (Deprez et al. 2018). In addition, Heinze and Weber (2016) find that intrapreneurs do not act on their own and that successful intrapreneurship depends on the employees’ ability to recruit others for their ideas. Freely accessible open workplaces create the opportunity for intrapreneurs to easily connect with likeminded individuals and pitch their ideas (Bouncken et al. 2018). Therefore, we expect that the facet freely accessible open workplaces is positively related to intrapreneurial behaviour.

#### H1e

Freely accessible open workplaces are positively related to intrapreneurial behaviour.

### The mediating role of co-worker social interaction

#### NWW and co-worker social interaction

Social interaction is central to social exchanges in any organisation; it shapes norms and values, and is at the basis of networks of employees that are cooperating to achieve a common goal (Cohen and Prusak 2001). Social interactions between employees are likely to change with the implementation of NWW, but the direction of the change is not a priori clear from existing evidence (Gerards et al. 2018).

The NWW facets time- and location-independent work, management of output and access to organisational knowledge contribute to extending communication beyond regular office hours and the physical office, and promote online communication instead of real life interaction. This could facilitate social interaction between co-workers as it provides employees with a broader range of communication options (Boswell and Olsen-Buchanan 2007; Diaz et al. 2012). These NWW facets also feature in the literature on flexible work design, teleworking or telecommuting (Gerards et al. 2018). For instance, Halford (2005) provides examples of how telework affects organisational relationships and interactions in positive as well as negative ways. Some other studies however, emphasise the positive effects of these work designs on the quality of co-worker communication (e.g. ten Brummelhuis et al. 2012; ter Hoeven and van Zoonen 2015).

Next, empirical evidence points towards a positive relationship between the fourth NWW facet, flexibility in working relations, and social interaction. For instance, Kossek and Lee (2008) find that working less hours because of a reduced workload, is positively related to communication between colleagues. In addition, Branine (2003) finds that workers in flexible work arrangements experience mutual support from their colleagues.

The open-plan offices central to the fifth NWW facet, freely accessible open workplace, are especially designed to foster social interactions and are therefore naturally related to social interaction (Bouncken and Reuschl 2018; Brunia et al. 2016; Gerards et al. 2018).

However, flexible work, intensive use of ICT and open-plan offices could also obstruct social interactions. As there is no designated place and time for employees to be available, colleagues might struggle to cooperate due to asynchronous and dispersed work habits. Moreover, working from home or elsewhere using ICT can also contribute to social isolation of professionals (Cooper and Kurland 2002; Kurland and Cooper 2002). Morganson et al. (2010) show for instance a negative relation between working outside the office and social interaction at work. Similarly, Kingma (2018) describes an example how some workers turned the option to work from home into a right; claiming a fixed day for home-working and therewith actually reducing their flexibility to be available for office-based meetings. Lastly, open-plan offices have downsides of loss of privacy and increased noise (Kim and de Dear 2013).

To our knowledge, the only direct analysis of NWW facets on social interaction, Gerards et al. (2018), finds that only access to organisational knowledge and freely accessible open workplaces are positively related to social interaction.

#### Co-worker social interaction and intrapreneurial behaviour

Research is more consistent concerning the relation between co-worker social interaction and employee intrapreneurship. Social interaction forms a key element of the intrapreneurial process as it is at the core of intrapreneurial networking activities. Organisational networks are essential for employees to gather and link new information and knowledge, and to find new opportunities for the organisation (Bjornali and Støren 2012; Hayton and Kelley 2006; Rigtering and Weitzel 2013). These networks also play a pivotal role in accessing and convincing superiors and key decision makers in the organisation of the value of the new opportunity, which paves the way for further development and implementation (Hayton and Kelley 2006). Thus, social interaction helps employees to create an appropriate network for developing an intrapreneurial idea (Castrogiovanni et al. 2011). In sum, social interaction is essential for intrapreneurs in order to be able to gather information, recognise opportunities and convince people in the organisation to support their initiative.

Based on the literature discussed above we hypothesise that the relation between the NWW facets access to organisational knowledge and freely accessible workplaces and intrapreneurial behaviour is partially but positively mediated by social interaction:

##### H2a

Co-worker social interaction is positively related to intrapreneurial behaviour.

##### H2b

Co-worker social interaction mediates the relation between NWW facets access to organisational knowledge and freely accessible open workplaces and intrapreneurial behaviour positively but only partially.

### The mediating role of transformational leadership

#### NWW and transformational leadership

NWW also affect social exchange relations between managers and their employees. As NWW entail management of output rather than work processes, and allow a considerable level of autonomy to employees, managers in the organisation need to adapt their management style accordingly (Gerards et al. 2018; Johnson 2004; Peters and Den Dulk 2003; Wright and Oldford 1993). Arguably, the introduction of NWW facets time- and location-independent work and access to organisational knowledge eliminates the need for managers to see their employees daily. Consequently, managers need to change from direct supervision to managing of output (de Leede and Krajenbrink 2014). As such, Peters et al. (2014) argue that the implementation of NWW prompts managers to show behaviours associated with transformational leadership.

Transformational leadership describes leaders who raise and broaden the interest of their followers. They provide direction and a vision to work towards common goals, and inspire and motivate employees to aim for successful outcomes. Transformational leaders are charismatic, provide intellectual stimulation and have consideration for the individual situation of the employee (Bass 1999). They manage their teams by setting objectives and giving frequent feedback which improves motivation, satisfaction and performance (Hertel et al. 2005). Therefore, transformational leadership entails positive social exchange relationships between managers and their employees.

Several studies find that transformational leadership mediates between NWW and employee level outcomes. For instance, de Leede and Kraijenbrink (2014) find that trust—which is an essential element of transformational leadership and an outcome of positive social exchange relationships (Tanghe et al. 2010)—mediates the relation between NWW and employee performance. Similarly, Gerards et al. (2018) find that transformational leadership positively mediates the relation between NWW facets time and location independent work, access to organisational knowledge, and freely accessible open workplaces and work engagement. Importantly, in testing transformational leadership as a moderator (as opposed to a mediator), de Leede and Heuver (2016) do not find a significant effect of managers’ transformational leadership qualities on the relation between NWW and organisational commitment. Therefore, following Gerards et al. (2018) and de Leede and Kraijenbrink (2014), we include transformational leadership as mediator between the NWW facets and our employee level outcome, intrapreneurial behaviour.

#### Transformational leadership and intrapreneurial behaviour

Mutual trust and obligations are created as the relationship quality between managers and employees progresses to transformational qualities (Graen and Uhl-Bien 1995). These positive exchange relationships between employees and their managers stimulate employees to engage in innovative activity that goes beyond role prescriptions (Bammens 2016; Hughes et al. 2018). We therefore expect that employees may reciprocate the positive social exchange relations associated with transformational leadership with intrapreneurial behaviour. In addition, transformational leaders communicate clear visions that may guide employees in recognising opportunities for their organisations and stimulate employees in coming up with new ideas (Howell and Higgins 1990; Moriano et al. 2014). Furthermore, leaders play an important role in facilitating employees’ new ideas (Deprez et al. 2018; Heinze and Weber 2016), for instance by creating an atmosphere of trust in which employees feel comfortable to share their innovative ideas (Krawczyk-Sokolowska et al. 2019). Moreover, new initiatives often go against the status quo in an organisation and are therefore likely met with resistance from other organisational members (Deprez et al. 2018; Heinze and Weber 2016). Transformational leaders provide employees with the leverage needed to overcome organisational boundaries and to find organisational support for their ideas (Rosing et al. 2011).

Indeed, empirical evidence shows that transformational leadership positively relates to intrapreneurial behaviour (Moriano et al. 2014). Moreover, Kuratko et al. (2014) show that the encouragement and empowerment that a transformational leader provides coincides with the management support intrapreneurs need, and Deprez and Euwema (2017) show that intrapreneurs expect their managers to portray transformational leadership behaviours. They find that intrapreneurs expect their managers to provide, for instance, autonomy, trust, a personal connection and sufficient feedback. Based on the discussed literature above, we hypothesise that transformational leadership positively but partially mediates the relation between all NWW facets and intrapreneurial behaviour:

##### H3a

Transformational leadership is positively related to intrapreneurial behaviour.

##### H3b

Transformational leadership positively mediates the relation between all NWW facets and intrapreneurial behaviour partially.

## Methods

### Data and sample

We developed a survey for the purpose of this study. In the spring of 2018, we approached 13,665 members of the Dutch population via an online panel.^5^ 629 individuals responded positively to the request to partake in the survey. Of these 629, only those who indicated to be in paid employment were routed to continue the survey. This led to the exclusion of 329 respondents not in paid employment. In total 300 individuals completed the survey. For our analyses, we excluded 46 participants who were older than 67 years—which is the official retirement age in the Netherlands—or indicated not to have a manager or supervisor or not to work in a building.

Our final sample consists of 254 individuals working in 13 different economic sectors.^6^ Table 1 shows summary statistics of our sample and variables. The mean age of our sample is 49.3 years (SD = 10.5) and the mean tenure is 14.5 years (SD = 11.5). Furthermore, 44.1% is female, and 43.3% of our total sample works part-time. Moreover, more than half of individuals in our sample is higher educated (53.94%) and 27.95% of individuals work in a management or sales occupation.

**Table 1:** Sample and variable summary statistics

	Mean	SD	Min	Max
*Intrapreneurship*				
Strategic renewal behaviour	2.7	1.3	1.0	6.5
New venture behaviour	2.0	1.2	1.0	6.0
Total intrapreneurship	2.4	1.2	1.0	6.0
*NWW variables*				
Time- and location-independent work	2.2	1.2	1.0	5.0
Management of output	3.2	0.9	1.0	5.0
Access to colleagues	3.6	0.8	1.0	5.0
Access to information	3.3	1.1	1.0	5.0
Flexibility in working relations	2.8	1.0	1.0	5.0
Freely accessible open workplaces	3.5	1.1	1.0	5.0
*Mediating variables*				
Transformational leadership	3.1	1.1	1.0	5.0
Social Interaction	3.7	0.8	1.0	5.0
*Control variables*				
Neuroticism	2.6	0.8	1.0	4.7
Extraversion	3.4	0.7	1.7	5.0
Openness	3.2	0.7	1.3	5.0
Agreeableness	3.8	0.6	2.0	5.0
Conscientiousness	3.9	0.6	1.7	5.0
Age (in years)	49.3	10.5	25	67
Organisational tenure (in years)	14.5	11.5	0.1	45.8

	N	%
Female	112	44.1
Lower educational level	23	9.1
Middle educational level	94	37.0
Higher educational level	137	53.9
Part-time work	110	43.3
Management or sales occupation	71	28.0
Total N	254	

Summary statistics based on unweighted and unstandardized sum-scores for the items of each scale

### Measures

#### Employee intrapreneurial behaviour

We measure intrapreneurial behaviour using the Dutch version of the ‘Employee Intrapreneurship Scale’ (EIS; Gawke et al. 2019). The EIS consists of fifteen items measuring employees’ strategic renewal behaviours (eight items) and new venture behaviours (seven items). Example items are “I undertake activities to realise change in my organisation” and “I actively establish new collaborations with experts outside of my own expertise.” Respondents rated all items on a 7-point Likert scale ranging from 1 (Never) to 7 (Always). Cronbach’s alpha of the total scale is high (α = 0.97). We perform a polychoric factor analysis (Holgado-Tello et al. 2010) and subsequently calculate the total scale score by means of regression scoring. In addition, we standardise the total scale scores before our analyses.

#### New ways of working

Each NWW facet covers a single domain where the items that measure it are defining characteristics of the facet; a change in these items would also change the conceptual domain of the facet. This is indicative of the reflective properties of the NWW facets (Fleuren et al. 2018; Jarvis et al. 2003). However, each facet does not capture the same phenomenon as would be the case in a reflective construct (Fleuren et al. 2018; Jarvis et al. 2003), but rather covers a different independent aspect of NWW. Moreover, the NWW facets can have their own antecedents and effects. These characteristics are indicative of formative constructs (Fleuren et al. 2018; Jarvis et al. 2003). Therefore, we regard NWW as a reflective first order, formative (causal) second order construct (Fleuren et al. 2018; Jarvis et al. 2003), where each of the facets covers a different aspect of NWW.

We measure the extent to which the NWW facets are present in employees’ organisations by using and building on the NWW scale developed by Gerards et al. (2018). Their scale consists of five subscales measuring the five NWW facets of ‘time- and location-independent work’, ‘management of output’, ‘access to organisational knowledge’, ‘flexibility in working relations’, and ‘freely accessible open workplace’. However, their subscales for management of output and flexibility in working relations consist of only one item each. Moreover, Gerards et al. (2018) reported Cronbach’s α coefficients of 0.78 for their subscales for time- and location-independent work and freely accessible open workplace, and 0.77 for their subscale for access to organisational knowledge. While these scores are satisfactory, they provide room for improvement. Hence, to reduce the reliance on single-item subscales and in an effort to increase the reliability of the subscales, we chose to expand several of the original subscales as used in Gerards et al. (2018) with new items.^7^ “Table 3 in the Appendix” provides an overview of all items originating from Gerards et al. (2018) and the new items we added. Respondents were asked to indicate the degree to which each statement applies to their current job on a 5-point Likert scale ranging from 1 (not at all) to 5 (to a very high extent).

The results of a factor analysis, which are included in “Table 4 in the Appendix”, on the combination of all old and new items, indicate a six factor solution for our sample. We excluded one item (“I am able to set my own working hours”) of the facet time- and location-independent work that showed a cross-loading onto two factors with a loading lower than 0.2. The old and new items for facets 2 ‘management of output’, facet 4 ‘flexibility in working relations’ and facet 5 ‘freely accessible open workplaces’ all load onto their respective factors. In addition, the results of this analysis show that the items for facet 3 ‘access to organisational knowledge’ load onto two different factors representing access to organisational knowledge through colleagues on the one hand, and access to organisational knowledge through information systems on the other hand. Therefore, we decided to split NWW facet 3 into two separate facets: facet 3a ‘access to colleagues’ and facet 3b ‘access to information’.

The Gerards et al. (2018) subscale for the NWW facet time- and location-independent work consisted of two items, to which we added one new item (“I am able to work from home if I want to”). However, as described in the previous paragraph, we excluded the original Gerards et al. (2018) item “I am able to set my own working hours”. Therefore, we measure the NWW facet time- and location-independent with two items (α = 0.90).

The Gerards et al. (2018) subscale for the NWW facet management of output consisted of only one item “I am able to determine the way I work”. We added two new items that together more clearly delineate that management of output pertains to the social exchange relationship between supervisor and employee, and to the quality of the work and not to the way it has been achieved. These new items are “My supervisor does not get involved with the way I do my job” and “My supervisor evaluates me on the quality of the work I deliver, not the way I worked”. Cronbach’s alpha for these three items is 0.73, which is satisfactory, and could not be increased by omitting any of the items.

We added one new item (“I have access to all necessary information everywhere, at any time”) to the original Gerards et al. (2018) four item scale for the NWW facet access to organisational knowledge. Thereby, it now also accounts for the (lack of) restrictions in time at which knowledge can be accessed.^8^ As described above, these five items load onto two factors describing access to organisational knowledge through colleagues and information systems. Consequently, we decided to proceed our analyses with two variables capturing this distinction in knowledge access. We measure ‘access to colleagues’ with three items (α = 0.80), and ‘access to information’, with two items (α = 0.78).

Gerards et al. (2018)’s measurement of the subscale flexibility in working relations consisted of only one item “I have the ability to adapt my working scheme to my phase of life and ambitions”, to which we added one new item that adds a level of explicitness to the subscale (“I have the possibility to work more or fewer hours”). Cronbach’s alpha for these two items is sufficient (α = 0.69).

The NWW subscale freely accessible open workplaces consisted of two items to which we added one more item (“The building is arranged in such a way that I enjoy working there”). This three item subscale results in the same Cronbach’s alpha (α = 0.82) compared to when we use the original two item subscale. Therefore, we do not include this new item, and proceed our analyses using the original two items from Gerards et al. (2018).

We calculate a score for all NWW facets using regression scoring after a polychoric factor analysis (Holgado-Tello et al. 2010). Moreover, we standardise the total scale scores before our analyses.

#### Transformational leadership

We measure transformational leadership using 6 items from Carless et al. (2000). Respondents were asked to indicate the extent to which the six items apply to their supervisor on a 5-point Likert scale ranging from 1 (never) to 5 (always). Example items are “My supervisor communicates a clear and positive vision of the future” and “My supervisor treats staff as individuals, supports and encourages their development”. Cronbach’s alpha of the scale is high (α = 0.96).

#### Social interaction

We measure social interaction using the two statements from Gerards et al. (2018). One statement concerns the cooperation between colleagues (“I find working with my colleagues pleasant”). The other statement concerns the speed at which the respondent is helped by colleagues when he or she faces problems (“When facing problems, I quickly receive help from colleagues”). Respondents rated their agreement with these items on a 5-point Likert scale ranging from 1 (not at all) to 5 (to a very high degree). Cronbach’s alpha of the scale is satisfactory (α = 0.79).

#### Control variables

We include several individual level control variables. First, we control for employees’ education level. Previous studies show an ambiguous relation of education level with intrapreneurship. De Jong et al. (2015) and Urbano and Turró (2013) find a positive relation with intrapreneurship in a Dutch consultancy firm and in various sectors in nine European countries, respectively. However, Camelo-Ordaz et al. (2012), find a negative relation between education level and intrapreneurship in Spanish small creative firms. We construct a categorical variable indicating an employee’s lower (= 1), middle (= 2) or higher (= 3) education level.

Second, previous research indicates that older employees and those with longer organisational tenure are less involved in intrapreneurship (Camelo-Ordaz et al. 2012; Parker 2011). We therefore control for age and organisational tenure. Third, women appear to be less involved in intrapreneurship than men (Adachi and Hisada 2017; Parker 2011). Therefore, we include a gender dummy variable (1 if female). Fourth, Adachi and Hisada (2017) show that employees working part-time are less involved in intrapreneurship. We therefore include a dummy indicating part-time (= 1), i.e. a maximum of 32 h per week, or full-time employment (= 0), i.e. more than 32 h per week.

Last, we control for employees’ personality by including measures for all Big Five personality traits. Some earlier studies investigate the relationship between personality traits and intrapreneurship. These studies find that the Big Five traits openness to experience (Farruhk et al. 2016) and extraversion (Farruhk et al. 2016; Sinha and Srivastava 2013) are positively related to intrapreneurship. Contrarily, neuroticism (Sinha and Srivastava 2013), agreeableness (Farruhk et al. 2016) and conscientiousness appear to be negatively related to intrapreneurship. We use the 15-item Big-Five measure by Lang et al. (2011) that includes statements as “I see myself as extraverted, enthusiastic” and “I see myself as open to new experiences, complex”. All items are rated on a 5-point Likert scale ranging from 1 (completely disagree) to 5 (completely agree).

### Variable descriptives and a note on potential common method variance

Table 1 contains summary statistics of the variables in our study. Noteworthy are the relatively high mean on the facet access to organisational knowledge through colleagues and the relatively low means on the facets time- and location-independent work and flexibility inworking relations. This shows that access to organisational knowledge through colleagues is more widespread than the other NWW facets. In addition, the mean for intrapreneurial behaviour is quite low, indicating a skewed distribution of intrapreneurial behaviour scores in our sample. This is consistent with previous studies that find a low prevalence of intrapreneurial behaviour among employees (Bosma et al. 2010). Most individuals in our sample are highly educated (53.9%) and more than half are male (55.9%).

Table 2 shows the correlations between all variables. All NWW facets are positively correlated with intrapreneurial behaviour, transformational leadership and social interaction. In addition, both transformational leadership and social interaction are positively correlated with intrapreneurial behaviour.

**Table 2:** Correlations between main variables

	1	2	3a	3b	4	5	6	7
1. Time- & location-independent work	1.000							
2. Management of output	0.479***	1.000						
3a. Access to colleagues	0.301***	0.486***	1.000					
3b. Access to information	0.452***	0.500***	0.424***	1.000				
4. Flexibility in working relations	0.548***	0.537***	0.412***	0.432***	1.000			
5. Freely accessible open workplace	0.318***	0.297***	0.485***	0.343***	0.371***	1.000		
6. Intrapreneurial behaviour	0.376***	0.350***	0.201**	0.262***	0.232***	0.300***	1.000	
7. Transformational leadership	0.285***	0.394***	0.468***	0.223***	0.386***	0.468***	0.312***	1.000
8. Social interaction	0.173**	0.358***	0.564***	0.191**	0.332***	0.303***	0.145*	0.436***

*NWW* New ways of working

Correlations based on unweighted and unstandardized sum-scores for the items of each scale. *p < 0.05, **p < 0.01, ***p < 0.001

Using a single source for the measurement of our main variables could lead to common method variance (CMV), possibly causing inflated correlations between the variables (e.g. Podsakoff et al. (2003) for a seminal review on CMV and Fuller e al. (2016) for a recent discussion of its implications for business research). To assess whether we should be concerned with this we performed a Harman one-factor test, which entails an exploratory factor analysis on all items underlying all subjective variables in Table 1. This analysis did not produce one single factor that accounted for most of the variance between the variables. In fact, the results show that the first factor accounts for 39.9% of the total variance. Commonly, a percentage below 50% is considered a result that indicates lack of CMV, or at worst CMV small enough not to upwardly bias correlations (Fuller et al. 2016).

### Estimation methodology

We test our hypotheses using the multiple mediation bootstrap method by Preacher and Hayes (2008). This method uses OLS regressions to estimate all coefficients, and bootstrapping to determine the confidence intervals for the direct and indirect effects. As such, this method allows us to test the existence of indirect effects, which are only inferred when we would apply a causal steps approach (Baron and Kenny 1986; Hayes 2009).

## Results

### New ways of working facets and intrapreneurial behaviour

**Fig. 1:**
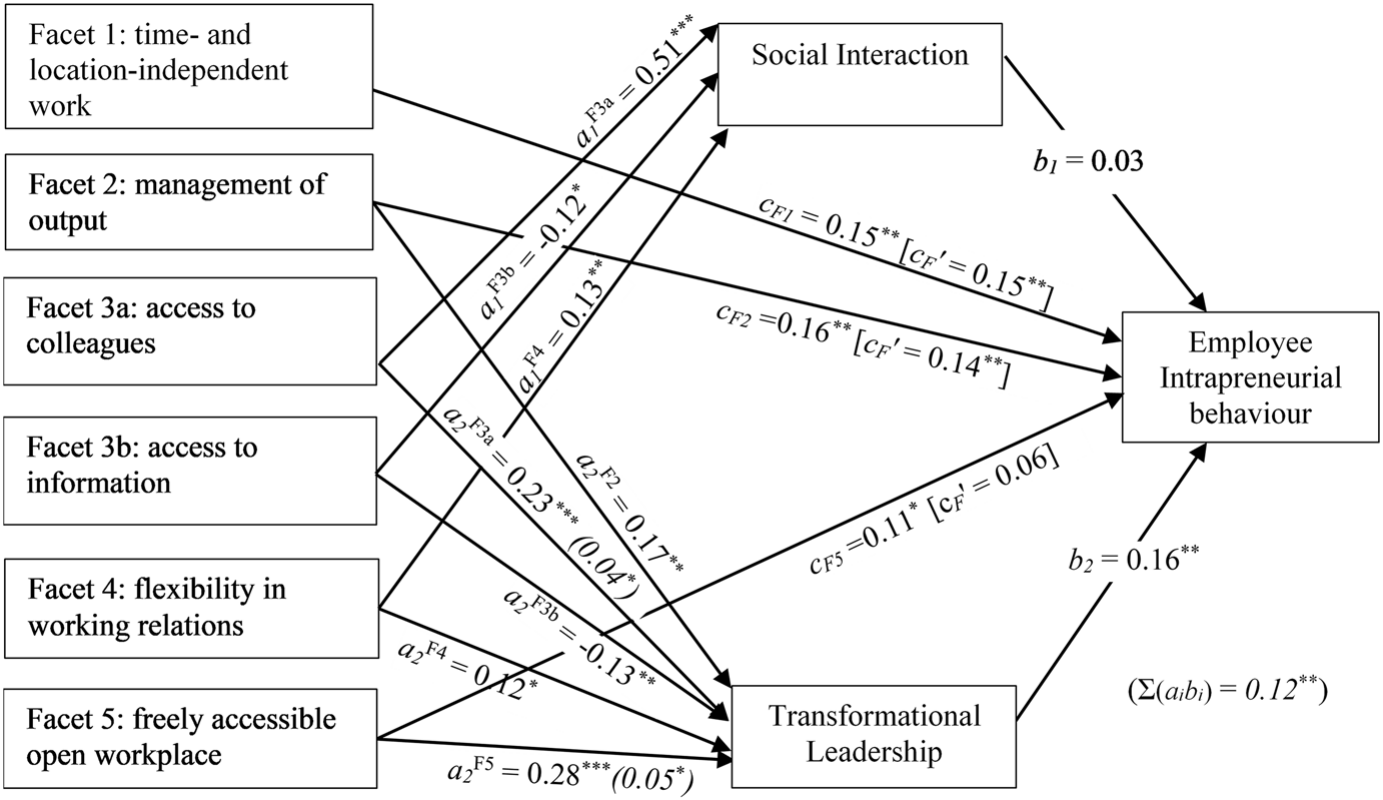
Multiple mediation model of direct and indirect effects of NWW facets on intrapreneurial behaviour.* Note* Indirect effects (aibi) and total indirect effect (Σ(aibi)) are shown in italics in parentheses. Direct effect of NWW facets accounting for mediation is shown in brackets. We show only significant relationships. Total effect per facet ‘F’ is cF = Σ(ai,F*bi) + cF’. *p<0.1, **p<0.05, ***p<0.01

Figure 1 shows the estimation results of the relations between the NWW facets and intrapreneurial behaviour. The results show that the facets time- and location-independent work (β = 0.15, *p* < 0.05), management of output (β = 0.16, *p* < 0.05) and freely accessible open workplaces (β = 0.11, *p* < 0.1) have a positive relation with intrapreneurial behaviour before accounting for mediation. The other NWW facets show no significant relation with intrapreneurial behaviour. Therefore, we find support for hypotheses 1a, 1b and 1e, but not for hypotheses 1c and 1d.

Next, we observe that the majority of NWW facets are related to transformational leadership and social interaction. The facets management of output (β = 0.17 *p* < 0.05), access to organisational knowledge through colleagues (β = 0.23, *p* < 0.01), flexibility in working relations (β = 0.12, *p* < 0.1) and freely accessible open workplaces (β = 0.28, *p* < 0.01) have a significant positive relation with transformational leadership. However, access to organisational knowledge through information systems is negatively (β = − 0.13, *p* < 0.05) related to transformational leadership, and time- and location-independent work is not related to transformational leadership. Furthermore, we find that access to organisational knowledge through colleagues (β = 0.51, *p* < 0.01) and flexibility in working relations (β = 0.13, *p* < 0.1 are positively related to social interaction. However, we find that access to organisational knowledge through information systems (β = − 0.12, *p* < 0.01) is negatively related to social interaction.

Of the expected mediators only transformational leadership (β = 0.16, *p* < 0.05) is significantly related to intrapreneurial behaviour.^9^ Therefore, we find support for hypothesis 3a, but not for hypotheses 2a and 2b. The mediating effect of transformational leadership renders the direct effect of freely accessible open workplaces on intrapreneurial behaviour insignificant after accounting for mediation (β = 0.06, *p* > 0.1). The indirect effect of freely accessible open workplaces *(*β = 0.05, *p* = 0.05) on intrapreneurial behaviour through transformational leadership is significant and accounts for 45.5% of the total effect of the freely accessible open workplace. Further, despite the absence of a direct effect of access to organisational knowledge through colleagues on intrapreneurial behaviour, we find a marginally significant indirect effect of this facet on intrapreneurial behaviour via transformational leadership (β = 0.04, *p* < 0.1). We find no other significant mediation effects and as a result the direct effects of the facets time- and location-independent work and management of output on intrapreneurial behaviour show little to no change in size or significance after accounting for mediation. Therefore, hypothesis 3b is only partially supported.

Furthermore, in line with the literature discussed in paragraph 3.2.5, we find that those in management or sales jobs and employees in full-time employment have significantly higher levels of intrapreneurial behaviour than employees in other types of jobs and those in part-time employment, respectively. In addition, openness to experience is positively related to intrapreneurial behaviour. However, contrary to our expectations, the other control variables have no significant relation with intrapreneurial behaviour.^10^ Altogether, the estimated model explains 40% of the variance in intrapreneurial behaviour and is highly significant (*p* < 0.01).

### Relationships for strategic renewal behaviour and new venture behaviour

**Fig. 2:**
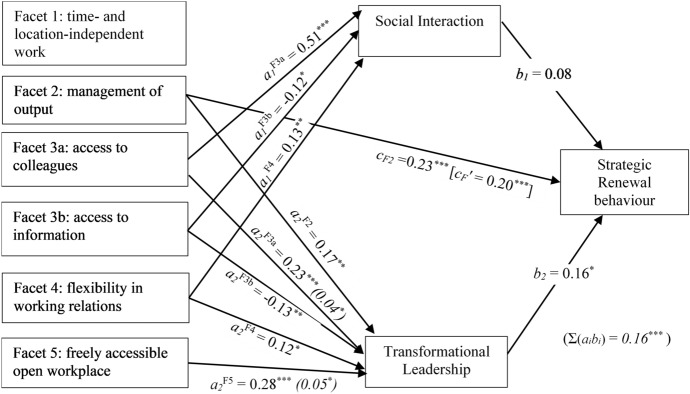
Multiple mediation model of direct and indirect effects of NWW facets on Strategic renewal behaviour. *Note:* Indirect effects (*a*_*i*_*b*_*i*_) and total indirect effect (Σ(*a*_*i*_*b*_*i*_)) are shown in italics in parentheses. Direct effect of NWW facets accounting for mediation is shown in brackets. We show only significant relationships. Total effect per facet *‘F’* is *c*_*F*_= Σ(*a*_i,F_^*^b_*i*_) + *c*_*F*_*’*. *p < 0.1, **p < 0.05, ***p < 0.01

**Fig. 3:**
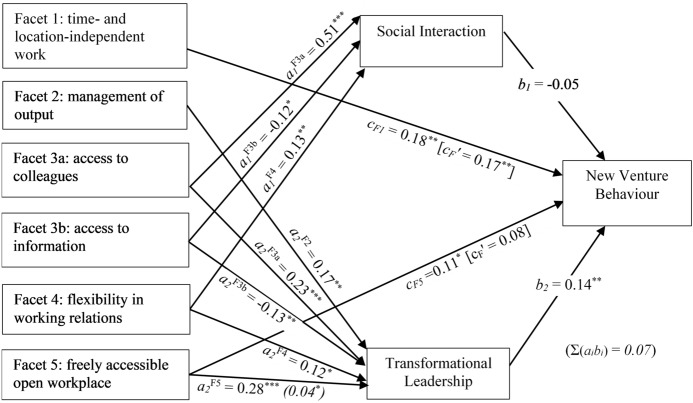
Multiple mediation model of direct and indirect effects of NWW facets on New Venture behaviour. *Note:* Indirect effects (*a*_*i*_*b*_*i*_) and total indirect effect (Σ(*a*_*i*_*b*_*i*_)) are shown in italics in parentheses. Direct effect of NWW facets accounting for mediation is shown in brackets. We show only significant relationships. Total effect per facet *‘F’* is *c*_*F*_= Σ(*a*_i,F_^*^b_*i*_) + *c*_*F*_*’*. *p < 0.1, **p < 0.05, ***p < 0.01

We further analysed if these results differ for the two subscales of intrapreneurial behaviour. “Figures 2 and 3 in the Appendix” show the results for the relations between the NWW facets and strategic renewal and new venture behaviour, respectively. Most noteworthy is that, after accounting for mediation, management of output is significantly related to strategic renewal behaviour (β = 0.20 *p* < 0.01), but not to new venture behaviour (β = 0.04, *p* > 0.1). Moreover, time- and location- independent work is positively related to new venture behaviour (β = 0.18, *p* < 0.05), but not to strategic renewal behaviour (β = 0.14, *p* < 0.05). In addition, we find positive relations between transformational leadership and both strategic renewal (β = 0.16, *p* < 0.1) and new venture behaviour (β = 0.14, *p* < 0.05). However, this relation is only marginally significant for strategic renewal behaviour.

## Discussion

This paper presents a first investigation into the relation between the NWW facets and intrapreneurial behaviour and the mediating effects of both co-worker social interaction and transformational leadership, while controlling for employees’ age, education level, organisational tenure, gender and personality. In sum, we find that three NWW facets positively relate to intrapreneurial behaviour: time- and location-independent work, management of output and freely accessible open workplaces. However, while the facet time- and location-independent work is positively and significantly related to intrapreneurial behaviour, it is not related to the mediators. The facet management of output is positively related to both intrapreneurial behaviour and the mediator transformational leadership. However, as there is no significant indirect effect of management of output via transformational leadership, the coefficient of management of output on intrapreneurial behaviour hardly drops after accounting for the mediators. Next, we find that the relation between a freely accessible open workplace and intrapreneurial behaviour is fully mediated by transformational leadership. Although these relationships are positive, the estimated coefficients are small in magnitude. Therefore, these results give a first indication of the relationships between the NWW facets and intrapreneurial behaviour, but future research is needed to investigate the consistency and magnitude of these relationships.

Contrary to our expectations, we find no relation between co-worker social interaction and employees’ level of intrapreneurial behaviour. Thus, co-worker social interaction does not mediate between the facets of NWW and intrapreneurial behaviour. This unexpected result could be explained by the relatively focused way we measure co-worker social interaction. The measure we apply concerns the speed at which employees are helped by colleagues, and how pleasant employees experience the cooperation with their colleagues. While these are important aspects of social interaction for intrapreneurial behaviour, these do not directly incorporate the quality or content of social interactions between colleagues. Nevertheless, these findings suggest that horizontal social exchanges between colleagues play a lesser role in intrapreneurial behaviour as compared to vertical social exchanges between employees and supervisors. This is in line with previous research that points towards the key role of supervisors and middle managers in facilitating employees’ intrapreneurial behaviour (e.g. Edú Valsania et al. 2016; Deprez et al. 2018; Heinze and Weber 2016; Hornsby et al. 2002, 2009; Moriano et al. 2014).

Additional analyses show that the relationships of some NWW facets differ between the sub-aspects of intrapreneurial behaviour, and that some NWW facets even have negative relationships with sub-aspects of intrapreneurial behaviour. The facet management of output positively affects the strategic renewal behaviour aspect of intrapreneurial behaviour, but not the new venture behaviour aspect. They also show that time- and location-independent work positively affects new venture behaviour, but not strategic renewal behaviour. In addition, the positive relation between transformational leadership and intrapreneurial behaviour, and the mediation of transformational leadership between freely accessible open workplaces and intrapreneurial behaviour seems to be mainly driven by the new venture behavioural aspect of intrapreneurial behaviour. In addition, although not statistically significant, access to information through colleagues and flexibility in working relations both show negative signs in their relationship with intrapreneurial behaviour, suggesting that not all NWW facets stimulate intrapreneurial behaviour. These findings indicate that the relationships between NWW and intrapreneurial behaviour are complex, and depend not only on the extent to which specific NWW facets are implemented in an organisation, but also on the aspect of intrapreneurial behaviour under investigation.

### Contributions to the literature

This study provides a first investigation into the relation between the NWW facets and intrapreneurial behaviour. As such, we add to both the literature on the organisational antecedents of employees’ intrapreneurial behaviour and to the literature on employee outcomes of NWW. We add to the stream of literature on the organisational antecedents of employees’ intrapreneurial behaviour (e.g. de Jong et al. 2015; Kuratko et al. 2014; Rigtering and Weitzel 2013) by showing that several facets of NWW are positively related to intrapreneurial behaviour. Particularly, our finding that time- and location- independent work and management of output positively relate to intrapreneurial behaviour—for our sample of employees from 13 different sectors in the Netherlands—strengthens the finding of de Jong et al.’s (2015) single firm study that job autonomy is particularly important for stimulating employee’s intrapreneurial behaviour. Thus, these NWW facets could be added to the ways in which organisations can influence employee intrapreneurship (de Jong et al. 2015; Rigtering and Weitzel 2013). However, we find small effects sizes and differential effects depending on the aspect of intrapreneurial behaviour. Therefore, future research is needed to further explore the complexities, magnitude and the consistency of the relationship of NWW facets with intrapreneurial behaviour.

In addition, we contribute to the stream of literature that investigates the role of middle managers and leadership in intrapreneurship and intrapreneurial behaviour (e.g. Edú Valsania et al. 2016; Deprez et al. 2018; Heinze and Weber 2016; Hornsby et al. 2002, 2009; Moriano et al. 2014). Our study suggests that horizontal exchanges between co-workers are less important for intrapreneurial behaviour than the vertical social exchanges between managers and their employees. More specifically, our results suggest that the social exchanges between managers and their employees are important for stimulating intrapreneurial behaviour, especially for those behaviours aimed at the creation of new ventures for the existing organisation.

Moreover, we add to the stream of literature that investigates the individual level drivers of employees’ intrapreneurial behaviour (e.g. Hayton and Kelley 2006; Sinha and Srivastava 2013). Our study adds to an increased understanding of the influence of personality traits above and beyond the effects of organisational and managerial factors on intrapreneurial behaviour (e.g. Sinha and Srivastava 2013). We find that openness to experience positively relates to intrapreneurial behaviour, whereas the other Big Five traits do not.

Furthermore, we add to the literature on intrapreneurial behaviour by applying an alternative conceptualisation of intrapreneurial behaviour. Most previous studies that investigate individual and organisational drivers of intrapreneurial behaviours of employees (e.g. de Jong et al.), conceptualise intrapreneurial behaviours as innovative, proactive and risk taking behaviours. However, in the current study we have conceptualised intrapreneurial behaviour as behaviours aimed at new venture creation and strategic renewal (Gawke et al. 2019).

We also contribute to the literature on the effects of NWW on employee outcomes, such as Peters et al. (2014), de Leede and Kraijenbrink (2014), de Leede and Heuver (2016), Gerards et al. (2018, forthcoming) who find positive relations between NWW and work-related flow, employee performance and productivity, work engagement, and informal learning, respectively. In fact, work engagement could provide an interesting perspective for further investigating the relation between NWW and intrapreneurial behaviour, as Gawke et al. (2017) find evidence for a positive and cyclical relation between employees’ intrapreneurial behaviour, personal resources and work engagement. Implementing NWW practices may provide a way to instigate this positive gain cycle.

In addition, we contribute to the literature on NWW by refining the measurement of the NWW facets. First, we increase the measurement reliability of several facets, mostly by expanding the previously one-item facets of Gerards et al. (2018) to multiple-item facets. Second, the addition of new items and statistical testing lead to the division of the third NWW facet ‘access to organisational knowledge’ into two facets. These now make the distinction between access to organisational knowledge through information systems and access to organisational knowledge through colleagues. Importantly, these two facets show opposing signs in their relation to social interaction, transformational leadership, and—albeit not statistically significant—intrapreneurial behaviour. From the perspective of SET these results are interesting, because they suggest that the implementation of technology that enables access to organisational information may negatively affect the social exchange relationships between colleagues, and between employees and their managers. Therefore, future research on NWW is advised to make this distinction, too, as these opposing relations to other variables might otherwise cancel out against each other. However, as discussed below, more research is needed to develop a more reliable measurement of the NWW facets.

### Practical implications

Our results are particularly valuable in light of the recent COVID-19 induced massive increase in employees working from home (e.g. Brynjolfsson et al. 2020) with the support of ICT systems. Our results give rise to the suggestion that the recent increase in working from home could lead to an increase of employees’ intrapreneurial behaviour. Specifically, our study shows that the higher workers rate their job on the NWW facets time- and location- independent work and management of output—which are quintessential for working from home—the higher is their intrapreneurial behaviour. Interestingly, the relation of these two facets to intrapreneurial behaviour is virtually independent of organizational support in terms of transformational leadership and social interaction. But also regardless of the current COVID-19 triggered situation, our study shows that giving employees the autonomy to work independent of time and location and to determine their own work processes relates positively to intrapreneurial behaviour, which is useful information for organizations that want to stimulate intrapreneurial behaviour. Moreover, the positive relation between our mediator transformational leadership and intrapreneurial behaviour confirms that transformational leadership in itself also promotes intrapreneurial behaviour (see also Deprez and Euwema (2017) and Moriano et al. (2014)). In addition, our results show that a freely accessible open workplace only contributes to intrapreneurial behaviour if facilitated by a transformational leadership style. Therefore, organisations should consider actively promoting a transformational leadership style when they aim to foster employees’ intrapreneurial behaviour.

However, not all NWW facets contribute to fostering intrapreneurial behaviour. Our results suggest that (digitally) having access to organisational knowledge via colleagues or ICT systems does not necessarily support employees’ intrapreneurial behaviour. Specifically, our study shows that the NWW facets access to organisational knowledge through colleagues and through digital information sources and flexibility in working relations do not positively relate to intrapreneurial behaviour. This suggests that investments in ICT to allow for access to organisational knowledge should be carefully considered when an organisation aims at fostering employees’ intrapreneurial behaviour. Similarly, providing employees with the opportunity to adjust their contractual working hours does not seem an effective strategy for fostering intrapreneurial behaviour.

### Limitations and future research

The main limitation of this study is that we use cross-sectional data, which makes causal inferences about the relations in our study problematic. For instance, although the literature suggests that NWW facilitate intrapreneurial behaviour (e.g. Martins and Terblanche 2003), organisations with NWW might also be particularly attractive for intrapreneurial employees, i.e., intrapreneurs could self-select into firms that implemented NWW, and already display high levels of transformational leadership.

In addition, the use of cross sectional data could induce common method variance in our analyses. However, statistical test did not point towards the presence of common method variance. These test results, although never airtight, give us a good level of confidence in this regard. Further, the amount of control variables we include in our analyses is limited. Therefore, the estimated coefficients could be overestimated. The results of this study should therefore be interpreted with caution. Future studies should therefore investigate the consistency of the relationships of the NWW facets with intrapreneurial behaviour by implementing methods that avoid the potential for common method variance (Fuller et al. 2016; Podsakoff et al. 2003).

Moreover, using cross-sectional data implies that we cannot take account of the fact that NWW include facets that probably require time to become embedded after implementation. Similarly, the level of intrapreneurial behaviour is hardly expected to change overnight with the introduction of NWW practices. To investigate the causal effects of NWW on intrapreneurial behaviour, future research could therefore use longitudinal data or field experiments where the introduction of NWW and the effects on intrapreneurial behaviour are observed over time. In doing so, future studies could also further investigate differential effects of NWW on employees’ strategic renewal behaviour and new venture behaviour.

Besides taking time to be implemented, firms could also choose to implement different combinations of the NWW facets. However, we do not account for this in our analysis. It may be the case that certain combinations of NWW facets particularly stimulate intrapreneurial behaviour. Our results show that not all NWW facets are positively related to intrapreneurial behaviour, suggesting that the NWW facets do not always operate in unison with respect to intrapreneurial behaviour. Therefore, there may even be combinations of NWW facets that particularly hinder intrapreneurial behaviour. In addition, the optimal configuration of NWW facets that lead to intrapreneurial behaviour might depend on characteristics of firms, employees, culture and other contextual characteristics. For instance, there might be a different effect of specific combinations of NWW facets on intrapreneurial behaviour depending on the extent to which team work and autonomous project management are applied within specific industries. Therefore, future research could adopt a configurational approach (e.g. Harms et al. 2009) when studying the relationship between NWW and intrapreneurial behaviour.

Next, although we made improvements on the NWW facets measurement of Gerards et al. (2018), further research is needed to develop the measurement of NWW. This need is reflected for instance when comparing our results with the results of Gerards et al. (2018). Comparing both studies’ results, we observe inconsistent relationships between the NWW facets and transformational leadership and social interaction. This discrepancy could be due to either a sample bias in the current study or Gerards et al.’s (2018) study, or by the differences in the measurement of the NWW facets. If due to the latter, this would imply that the inclusion and exclusion of a few items in the way the NWW facets are measured matter for the conclusions we draw about observed relationships. Therefore, future efforts that enhance the measurement of the NWW facets are welcome.

Lastly, our results suggest that vertical social exchanges between employees and their supervisors play a more important role in facilitating intrapreneurial behaviour, than horizontal social exchanges between colleagues. However, our measure of social interaction does not measure the quality of social exchanges between colleagues. Therefore, future studies could investigate how NWW affects the *quality* of social exchanges between colleagues within teams, and how this in turn affects intrapreneurial behaviour. In addition, Hornsby et al. (2009) show that the positive relationship between work discretion and intrapreneurial behaviour is more positive for senior and middle level managers than it is for first-level managers. Therefore, it would be interesting to investigate the dynamic relations of NWW facets with intrapreneurial behaviour and social exchanges across hierarchical levels.

## Appendix

See Tables 3, 4, 5 and 6 and Figs. 2 and 3.

**Table 3:** New ways of working scale: original and new items

**Facet 1: time- and location-independent work**
1. I am able to set my own working hours
2. I am able to determine where I work
*3. I am able to work from home if I want to*
**Facet 2: management of output**
4. I am able to determine the way I work
*5. My supervisor does not get involved with the way I do my job*
*6. My supervisor evaluates me on the quality of the work I deliver, not the way I worked*
**Facet 3: access to organisational knowledge** **3a. Access to colleagues**
7. I am able to reach colleagues within the team quickly
8. I am able to reach managers quickly
9. I am able to reach colleagues outside the team quickly
**3b. Access to information**
10. I can access all necessary information on my computer, smartphone, and/or tablet
*11. I have access to all necessary information everywhere, at any time*
**Facet 4: flexibility in working relations**
12. I have the ability to adapt my working scheme to my phase of life and ambitions
*13. I have the possibility to work more or fewer hours*
**Facet 5: freely accessible open workplaces**
14. The building is arranged so that colleagues are easily accessible
15. The building is arranged so that managers are easily accessible

Newly added items in italics. Items that were excluded after factor analysis are indicated in strikethrough

**Table 4:** Results of factor analysis of all NWW items

NWW items	Factor 1	Factor 2	Factor 3	Factor 4	Factor 5	Factor6
**Facet 1**						
1. I am able to set my own working hours						
2. I am able to determine where I work		0.8408				
*3. I am able to work from home if I want to*		0.8613				
**Facet 2**						
4. I am able to determine the way I work	0.5747					
*5. My supervisor does not get involved with the way I do my job*	0.7067					
*6. My supervisor evaluates me on the quality of the work I deliver, not the way I worked*	0.4733					
**Facet 3**						
7. I am able to reach colleagues within the team quickly			0.8087			
8. I am able to reach managers quickly			0.6311			
9. I am able to reach colleagues outside the team quickly			0.6036			
10. I can access all necessary information on my computer, smartphone, and/or tablet				0.6683		
*11. I have access to all necessary information everywhere, at any time*				0.6995		
**Facet 4**						
12. I have the ability to adapt my working scheme to my phase of life and ambitions						0.4228
*13. I have the possibility to work more or fewer hours*						0.4717
**Facet 5**						
14. The building is arranged so that colleagues are easily accessible					0.7744	
15. The building is arranged so that managers are easily accessible					0.7743	

Rotated results (oblique oblimin). Blanks represent loadings < 0.3. Strikethrough indicates deleted item

**Table 5:** Mediation effect of NWW facets on intrapreneurial behaviour via social interaction and transformational leadership

Indirect effects	Coefficient	SE	Z	*p* value	95% Conf. interval	
					Lower	Upper
Facet 1 via Social Interaction (a_1_b_1_)	− 0.0016	0.0063	− 0.25	0.805	− 0.0139	0.0108
Facet 2 via Social Interaction (a_1_b_1_)	0.0018	0.0069	0.26	0.794	− 0.0117	0.0153
Facet 3a via Social Interaction (a_1_b_1_)	0.0132	0.0345	0.38	0.702	− 0.0544	0.0808
Facet 3b via Social Interaction (a_1_b_1_)	− 0.0030	0.0087	− 0.35	0.728	− 0.0200	0.0140
Facet 4 via Social Interaction (a_1_b_1_)	0.0035	0.0102	0.34	0.733	− 0.0165	0.0234
Facet 5 via Social Interaction (a_1_b_1_)	0.0016	0.0065	0.24	0.811	− 0.0112	0.0143
Facet 1 via Transformational Leadership (a_2_b_2_)	0.0014	0.0120	0.12	0.907	− 0.0221	0.0249
Facet 2 via Transformational Leadership (a_2_b_2_)	0.0272	0.0169	1.61	0.107	− 0.0059	0.0602
Facet 3a via Transformational Leadership (a_2_b_2_)	0.0362	0.0205	1.76	0.078	− 0.0041	0.0765
Facet 3b via Transformational Leadership (a_2_b_2_)	− 0.0201	0.0157	− 1.28	0.200	− 0.0509	0.0106
Facet 4 via Transformational Leadership (a_2_b_2_)	0.0187	0.0147	1.27	0.204	− 0.0102	0.0475
Facet 5 via Transformational Leadership (a_2_b_2_)	0.0453	0.0231	1.96	0.050	0.0000	0.0907
Total indirect effect	0.1241	0.0575	2.16	0.031	0.0113	0.2368
*Total effects*						
Total effect NWW facet 1	0.1509	0.0724	2.08	0.037	0.0090	0.2928
Total effect NWW facet 2	0.1647	0.0712	2.31	0.021	0.0252	0.3041
Total effect NWW facet 3a	− 0.0236	0.0608	− 0.39	0.698	− 0.1429	0.0956
Total effect NWW facet 3b	0.0061	0.0614	0.10	0.921	− 0.1143	0.1265
Total effect NWW facet 4	− 0.0613	0.0666	− 0.92	0.357	− 0.1917	0.0692
Total effect NWW facet 5	0.1101	0.0666	1.65	0.099	− 0.0205	0.2406

*SI* social interaction, *TL* transformational leadership. Results based on 4.999 bootstrap samples

**Table 6:** OLS regression results of NWW facets on intrapreneurial behaviour

Main variables	
Location-independent work	0.151**
	(0.066)
Management of output	0.136**
	(0.068)
Access to colleagues	− 0.073
	(0.070)
Access to information	0.029
	(0.063)
Flexibility in working relations	− 0.083
	(0.068)
Freely accessible open workplace	0.063
	(0.062)
Social interaction	0.026
	(0.065)
Transformational leadership	0.160**
	(0.063)
*Control variables*	
Female (*ref. *=* male)*	− 0.106
	(0.125)
Age	− 0.006
	(0.006)
Management or sales job	0.563***
	(0.116)
Tenure	− 0.003
	(0.005)
Part-time (*ref. *=* full*-*time)*	− 0.262**
	(0.120)
Lower level education (=* ref.)*	
Middle level education	− 0.118
	(0.188)
Higher level education	0.227
	(0.190)
Neuroticism	0.051
	(0.057)
Extraversion	− 0.007
	(0.055)
Openness to experience	0.202***
	(0.053)
Agreeableness	− 0.001
	(0.056)
Conscientiousness	0.056
	(0.055)
Observations	254
R-squared	0.40

## Footnotes

1 Transformational leadership entails positive social exchanges between leaders and their followers (Graen and Uhl-Bien 1995), whereby leaders create trust, inspire and provide a vision and common goal for all employees to aim for (Bass 1999). Transactional leadership entails leaders who create clear structures and roles, carefully monitor mistakes and failures, and provide rewards when goals are reached (Avolio et al. 1999; Moriano et al. 2014).

2 We refer to Blanka (2018) and Neessen et al. (2018) for systematic reviews on individual level intrapreneurship.

3 https://news.microsoft.com/2005/05/19/gates-outlines-new-world-of-work-at-ceo-summit/.

4 See the discussion in Gerards et al. (2018, pp. 520) on the ambiguous evidence regarding teleworking on social exchange relationships.

5 Data collection has been performed by the Dutch research company Etil (https://www.etil.nl).

6 These sectors include Construction, Retail, Financial services, Wholesale, Hospitality, Industry, Primary sector, Person services, Transportation, Commercial services, Education, Social services and others.

7 As we needed to add items to the subscales of management of output and flexibility in working relations (to expand them beyond just single-item subscales), we also chose expansion as the strategy to attempt to increase the reliability for the other subscales (instead of refining the original ones). Moreover, we also chose adding instead of refining items to maintain consistent comparability with the original subscales used in Gerards et al. (2018, forthcoming).

8 For instance, some companies (such as Volkswagen) have started restricting mobile access to e-mail after work hours: https://www.bbc.com/news/technology-16314901.

9 See “Table 5 in the Appendix” for the bootstrap results that determine the significance of the indirect and total effects of our mediation analysis. Percentile, bias-corrected and bias-corrected accelerated bootstrap results do not significantly differ and are available upon request from the authors.

10 A full table of results including control variables is included in “Appendix Table 6”.
